# Oxidation levels of North American over-the-counter *n*-3 (omega-3) supplements and the influence of supplement formulation and delivery form on evaluating oxidative safety

**DOI:** 10.1017/jns.2015.21

**Published:** 2015-11-04

**Authors:** Stefan A. Jackowski, Azhar Z. Alvi, Abdur Mirajkar, Zahabia Imani, Yuliya Gamalevych, Nisar A. Shaikh, George Jackowski

**Affiliations:** 1 University of Saskatchewan, Saskatoon, SK, Canada; 2 Pivotal Therapeutics Inc., Woodbridge, ON, Canada; 3 University of Toronto, Toronto, ON, Canada

**Keywords:** PUFA, Peroxides, Anisidine, Total oxidation, *n*-3 Supplements, Omega-3 supplements, AOCS, American Oil Chemists’ Society, CRN, Council for Responsible Nutrition, GOED, Global Organization for EPA and DHA Omega-3s, IFOS, International Fish Oil Standards, OTC, over-the-counter, TOTOX, total oxidation

## Abstract

The aim of the present study was to evaluate the oxidation status of North American *n*-3 (omega-3) PUFA nutritional supplements commercially available in Canada and evaluate the influence of product formulation and delivery form on oxidative safety. A total of 171 North American over-the-counter *n*-3 PUFA nutritional supplements were analysed for oxidation safety. Primary and secondary oxidation and total oxidation (TOTOX) were determined using the American Oil Chemists’ Society (AOCS) procedures. Comparisons between supplements’ final forms, oil source and *n*-3 PUFA concentration quartiles, as measures of product formulations and delivery forms, were compared using ANOVA. Of the products successfully tested, 50 % exceeded the voluntary recommended levels for markers of oxidation. Another 18 % of products were approaching the limits with 1–3 years before expiration. Encapsulated products without flavour additives had significantly lower secondary and TOTOX levels than bulk oils and flavoured products (*P* < 0·05). Children's products had significantly higher primary, secondary and TOTOX levels compared with all other products (*P* < 0·05). Markers of oxidation did not differ between oil sources (*P* > 0·05), with the exception of krill oil products having higher secondary oxidation levels than plant-based products (*P* > 0·05). Markers of oxidation did not differ between *n*-3 PUFA supplement concentration quartiles. Consumers may be at risk of exposure to higher levels of oxidative products. New regulatory mandates need to be introduced to ensure that all *n*-3 PUFA products, used as nutritional supplements, regardless of their formulation or delivery form, can be tested for oxidative safety and compliance.

An *n*-3 (omega-3) deficiency is a growing nutritional concern for many Western countries. The majority of individuals in Western countries fail to adequately meet daily nutritional requirements and, in Canada alone, over 55 % of individuals fail to consume the recommended one to two servings of fish per week^(^^1^^,^^2^^)^. As a result, many individuals compensate for their nutritional inadequacies with over-the-counter (OTC) nutritional supplementation as is shown by the sharp rise of supplement use over the last decade in industrialised nations^(^^3^^–^^5^^)^. Included in these nutritional supplements are *n*-3 products containing the essential long-chain *n*-3 PUFA EPA and DHA. EPA and DHA not only alleviate *n*-3 deficiency but have been widely recognised for their potential to reduce the risk of CVD, influence inflammatory cytokine response and improve cognitive performance^(^^6^^–^^12^^)^. Progressively, *n*-3 PUFA intake as a nutritional supplement is on the rise, as it is perceived as an easy, effective and safe method to alleviate an *n*-3 PUFA deficiency and achieve a number of associated health benefits. Additionally, children and adolescents represent a large proportion of vitamin and mineral supplementation users, with data from the USA indicating that nearly 35 % of youths between 2 and 17 years of age reported using nutritional supplements^(^^13^^)^. However, *n*-3 PUFA are chemically unstable and rapidly oxidise into primary lipid hydroperoxides and secondary oxidation products^(^^14^^,^^15^^)^, thereby triggering potential consumer safety issues with long-term exposure. Oxidation is an accelerated chain reaction, where small amounts of peroxides in the source oils or exposure to oxidising conditions may dramatically influence the rate of oxidation of *n*-3 PUFA. Furthermore, some *n*-3 PUFA products contain camouflaging or deodorising agents to make them more palatable which may further accelerate secondary oxidation^(^^14^^,^^15^^)^. Recent human studies have suggested that the levels of oxidisation can negatively affect the efficacy of an *n*-3 PUFA supplement, limiting the beneficial effects on circulating TAG and cholesterol levels^(^^16^^)^. Similarly, animal models provide compelling evidence that suggest the chronic exposure to oxidised *n*-3 PUFA may lead to growth retardation, increased inflammation, cardiomyopathy and carcinogenesis^(^^17^^,^^18^^)^. Thus, the levels of oxidation may dramatically affect the efficacy and safety of using *n*-3 PUFA nutritional supplements, which may counteract the intended use of these products. The exposure to oxidation may be additionally detrimental for growing children and adolescents since this is a unique period where rapid changes in growth and development are taking place. Investigations on the oxidative status of *n*-3 PUFA are therefore warranted to examine the oxidative safety of these commercially available products as nutritional supplements.

The oxidative status of *n*-3 PUFA supplements can be assessed through the determination of primary (peroxide; PV), secondary (anisidine; AV) and total oxidation (TOTOX) values (2 × PV + AV). Although there is currently no North American agency regulating the oxidation levels for all *n*-3 PUFA supplements containing EPA and/or DHA, voluntary standards for manufacturers have been proposed by international organisations endorsing peroxide, anisidine and TOTOX values of ≤ 5 mEq/kg, ≤20 and ≤26, respectively, as maximal limits for manufacturers of fish oil supplements^(^^19^^–^^22^^)^. These current standards are set for fish oil products only and are solely applicable to fish oil as an independent ingredient. As a result, previous studies investigating oxidation status have focused on fish oil only *n*-3 PUFA products, having documented high variability in their oxidation status^(^^23^^–^^29^^)^; however, the literature has consistently documented that oxidation levels in OTC fish oil nutritional supplements often exceed the current proposed voluntary recommended safety limits^(^^19^^–^^22^^)^. A recent study identified that 73 % of *n*-3 PUFA OTC fish oil supplements sold in South Africa were rancid and over 80 % had peroxide levels exceeding safety recommendations of ≤5 mEq/kg^(^^27^^)^. In the only study to assess both primary and secondary oxidation products, Albert *et al*.^(^^28^^)^ observed that of the thirty-two OTC *n*-3 PUFA supplements tested available in New Zealand and Australia, 83, 25 and 50 % exceeded the voluntary recommended safety limits for peroxide, anisidine and TOTOX, respectively. Though these seminal studies with fish oil *n*-3 PUFA indicated that there may be oxidation concerns in OTC supplements, they did not specifically address the potential impact of product formulation and delivery form on oxidative status. Since product handling and final formulation processing may introduce oxidising agents (e.g. air, light, O_2_) to the product's final consumer form it is important to consider the influence of product formulation (i.e. source oil, flavour additives, EPA and DHA content) and delivery form (i.e. encapsulation, direct bottling) on oxidative safety. There are no set standards for oxidative safety of *n*-3 PUFA in their final consumer form and the applicability of current testing methods on evaluating the oxidative products on other than fish oil *n*-3 PUFA source oils (e.g. krill, plant, seal, etc.) after product final formulation have not been considered or tested. Furthermore, with the shelf life of *n*-3 PUFA products being 3 years or more, the oxidative status may continue to deteriorate while products are still within the best before date. As such, the time to expiry warrants consideration when evaluating the oxidative safety of *n*-3 PUFA nutritional supplements. Therefore, the purpose of this study was (i) to evaluate the oxidation status of North American *n*-3 PUFA supplements, commercially available as OTC nutritional supplements in Canada, by determining peroxide, anisidine and TOTOX levels and, (ii) to further analyse these data to explore the influence of product formulation, delivery form and expiration on oxidative safety.

## Methods

### *n*-3 PUFA samples

A total of 171 *n*-3 PUFA supplements that were commercially available as over-the-counter supplements in Canada were analysed for this study. In an effort to provide a representative sample of Canadian *n*-3 PUFA supplements, any products advertised as an ‘omega-3 supplement’ were analysed. All products were purchased from local pharmacies and supermarkets in Newmarket, Ontario, Canada between April and November 2014. In all, forty-nine brands were tested and are presented in Table 1 in alphabetical order. Supplements were purchased in either softgel (e.g. capsule or minigel forms) or liquid (e.g. bulk oils) forms. Gummy supplements were excluded from analyses, as their composition and oxidation status could not be determined using the methods described below.

**Table 1.** Listing of the product names and brands tested (presented in alphabetical order)

1. A-vogel (Bioforce AG, Switzerland)	26. Newfoundland (David Health International, Canada)
2. Alterra Kids (Les Importations Herbsante Inc., Canada)	27. Nordic Naturals (Nordic Naturals Inc., Norway)
3. Bell (Bell Lifestyle Products Inc., USA)	28. Northern Seas (Northern Seas, Canada)
4. Benefishial (Pivotal Therapeutics Inc., Canada)	29. Norwegian Gold (Renew Life Canada Inc., Canada)
5. Cardio Strong (Platinum Naturals, Canada)	30. Now (Now Foods, Canada)
6. Carlson (J.R. Carlson Laboratories Inc., Norway)	31. Nu-Life (Nu Life North America, Canada)
7. David Health International (David Health International, Canada)	32. NutraSea (Ascenta Health, Canada)
8. Efamol (Flora Manufacturing, Canada)	33. OilSmart (Renew Life Canada Inc., Canada)
9. Flora (Flora Manufacturing, Canada)	34. Omega Factors (Natural Factors, Canada)
10. Garden of Life (Trophic Canada, Canada)	35. Organika (Organika Health Products Inc., Canada)
11. Genuine Health (Genuine Health, Canada)	36. Platinum (Platinum Naturals, Canada)
12. Green Pasture (Green Pasture Products, USA)	37. Prairie Naturals (Prairie Naturals, Canada)
13. Innovite Health (Inno-Vite Inc., Canada)	38. Progressive Nutritional Therapies (Progressive Nutritional Therapeutics, Canada)
14. iVita Omega-3 (Vita Health Products Inc., Canada)	39. Sea-licious (GAB Innovations, Canada)
15. Jamieson (Jamieson Laboratories, Canada)	40. Seven Seas (Merck, UK)
16. Kirkland Signature (WN Pharmaceuticals, Canada)	41. Sisu (Sisu Inc., Canada)
17. Kyolic (Purity Life Health Products, Canada)	42. Summit Supplements (Summit Supplements, Canada)
18. Minami Nutrition (Minami Nutrition, Belgium)	43. Sun Force (Sunforce International Products Inc., USA)
19. Naka (Naka Herbs and Vitamins Limited, Canada)	44. Swiss Natural (Swiss Herbal Remedies, Canada)
20. Natural Factors (Natural Factors, Canada)	45. Terra Nova (NuTran Furs Inc., Canada)
21. Naturally Organic (Naturally Organic Nova Scotia Organic Health Products Ltd, Canada)	46. TriStar Naturals (TriStar Naturals, Canada)
22. Nature's Harmony (Purity Life Health Products, Canada)	47. Trophic (Trophic Canada, Canada)
23. Nature's Way (Nature's Way of Canada, Canada)	48. Vitalux Plus (NutriCorp International, Canada)
24. New Chapter (New Chapter Inc., USA)	49. Webber Naturals (WN Pharmaceuticals, Canada)
25. New Roots Herbal (New Roots Herbal Inc., Canada)	

### Product groupings to evaluate product formulation and delivery form

Since product handling, processing and the inclusion of camouflaging or deodorising agents during the manufacturing processes may influence oxidation, supplements were categorised by formulation and delivery form criteria. Supplements were separated into four final consumer form groupings: (1) softgels unflavoured, consisting of *n*-3 PUFA supplements contained in a soft capsule with no added flavourings (*n* 47); (2) softgels with flavouring, consisting of *n*-3 PUFA supplements contained in a soft capsule with added flavours (*n* 62); (3) bulk oils, consisting of *n*-3 PUFA supplements bottled as oils with no encapsulation (*n* 33); and (4) children's products, consisting of *n*-3 PUFA supplements marketed specifically towards children (includes softgels, softgels with flavouring and bulk oils; *n* 29).

With the increasing diversity of *n*-3 PUFA supplements currently available in the Canadian market and the variety of sources from which these *n*-3 fatty acids are derived, supplements were also grouped by oil source. Of the *n*-3 PUFA nutritional supplements, 105 contained fish oils as their primary ingredient; eight contained krill as their primary ingredient; ten contained plant-based oils as their primary ingredient (e.g. flax seed); five contained other sources as their primary ingredient (e.g. seal and squid); and forty-three listed a combination of two or more sources.

### Oxidation analyses

All supplements were de-identified prior to all assessments. For each supplement, a 10 ml sample was extracted for peroxide and anisidine tests. Oils from encapsulated products were extracted from an incision at one end of the capsule and drawn into a clean, sterile, sealable, glass tube. The extracted oils from two or more capsules were combined to generate a pooled sample. All tubes containing oils were flushed with N_2_ gas, capped tightly and refrigerated prior to analyses. Peroxide and anisidine tests were performed within 30 min of extraction following the protocols outlined by the American Oil Chemists’ Society (AOCS) established procedures^(^^30^^,^^31^^)^. All peroxide and anisidine tests were performed in duplicate. Results from duplicate measures were within 10 %. Averages between duplicate measures are reported as peroxide and anisidine values. TOTOX was calculated from peroxide and anisidine measures using the formula TOTOX = ((2 × peroxide value) + anisidine value). All tests were performed by trained personnel at Pivotal Analytics (Woodbridge, Ontario, Canada).

### Statistical analyses

Data were checked for normality using skewness and kurtosis. No violations were observed; thus the supplement pool was accepted as being normally distributed. Outliers were removed from analyses if oxidation values exceeded ±3 sd. This resulted in the removal of one, six and four samples for peroxide, anisidine and TOTOX analyses, respectively. Comparisons between supplements grouped by final consumer form (softgels unflavoured, softgels flavoured, liquid bulk oils, children's products) and oil source (fish, krill, plant, other and combination) were performed using ANOVA with Tukey adjusted *post hoc* comparisons if significant group main effects were observed. To assess the influence of EPA + DHA concentration on oxidation status, the concentration of EPA and DHA for each supplementation was ascertained from the total amount of EPA + DHA per serving as indicated on the product labelling. Since serving sizes varied considerably between products, the concentration of EPA + DHA was recorded as the amount of EPA + DHA per 1000 mg for each supplement. For liquid products, 1 ml of product was assumed to be equivalent to 1000 mg for simplicity as most liquid emulsion products had label servings in tablespoon units. Supplements were then separated into concentration quartiles and comparisons between quartiles were assessed using ANOVA with Tukey adjusted *post hoc* comparisons if significant main effects were observed. Supplement peroxide, anisidine and TOTOX values were also compared against the international safety standards and recommendations established by the Global Organization for EPA and DHA Omega-3s (GOED), the Council for Responsible Nutrition (CRN) and International Fish Oil Standards (IFOS). Supplements were considered to have unacceptable oxidation levels if their peroxide, anisidine or TOTOX values exceeded 5 mEq/kg, 20 and 26, respectively. Additionally, products considered to have acceptable oxidation levels were subsequently evaluated to test if they were approaching these voluntary limits. A product was considered to be approaching the oxidation limits if its peroxide, anisidine and TOTOX values were higher than 75 % of the recommended international safety standards (i.e. peroxide values between 3·75 and 5 mEq/kg, anisidine values between 15 and 20, and TOTOX values between 19·5 and 26). An *α* of *P* < 0·05 was considered significant. All analyses were performed using Graph Pad Prism (Prism 6.0 for Mac OS X; Graph Pad) and data are presented as means and standard deviations unless otherwise stated.

## Results

Of the 171 *n*-3 PUFA supplements collected for analysis, 152 were observed to contain some form of antioxidant ingredient. This included additives such as tocopherols, vitamin E and rosemary extract. Peroxide and anisidine levels could not be determined for thirty-one and five *n*-3 PUFA nutritional supplements, respectively, due to the interference in end-point determination. Since TOTOX is calculated from peroxide and anisidine values, the TOTOX values for thirty-three supplements could also not be calculated. As a result, 139, 160 and 134 supplements were successfully tested for peroxide, anisidine and TOTOX from the 171 supplements collected. A breakdown of supplement final numbers is shown in Table 2. Table 3 displays the peroxide, anisidine and TOTOX values for all *n*-3 PUFA supplements that were successfully determined and identifies the number of supplements that were below the safety standards recommended by the GOED, CRN and IFOS. As seen in Table 1, 17 % of the supplements had peroxide values above 5 mEq/kg; 41 % had anisidine values above 20, and 39 % had TOTOX values above 26. Additionally, 50 % of all the products successfully tested failed at least one of the oxidation (e.g. peroxide, anisidine or TOTOX) recommended safety limits.

**Table 2.** Description of the final sample derived from the 171 over-the-counter *n*-3 products collected (*n*)

Oxidation assessment	Products collected	Products indetermined	Products successfully tested	Exceeded ±3 sd	Final numbers
Peroxide	171	31	140	1	139
Anisidine	171	5	166	6	160
TOTOX	171	33	138	4	134

TOTOX, total oxidation.

**Table 3.** Peroxide, anisidine and total oxidation (TOTOX) values for over-the-counter *n*-3 PUFA supplements and the number of supplements that failed international voluntary safety limits (Mean values and standard deviations)

	Descriptive			Exceeded limits*	
	*n*	Mean	sd	*n*	% of total
Peroxide (mEq/kg)	139	4·01	4·37	24	17
Anisidine	160	29·25	33·06	66	41
TOTOX	134	37·39	37·65	52	39

* Number of products that exceeded the international voluntary safety recommendations for peroxide (values >5 mEq/kg), anisidine (values >20) and TOTOX (values >26)^(^^19^^–^^22^^)^.

### Delivery form and formulation comparisons

#### Final consumer form comparisons

**Fig. 1.**
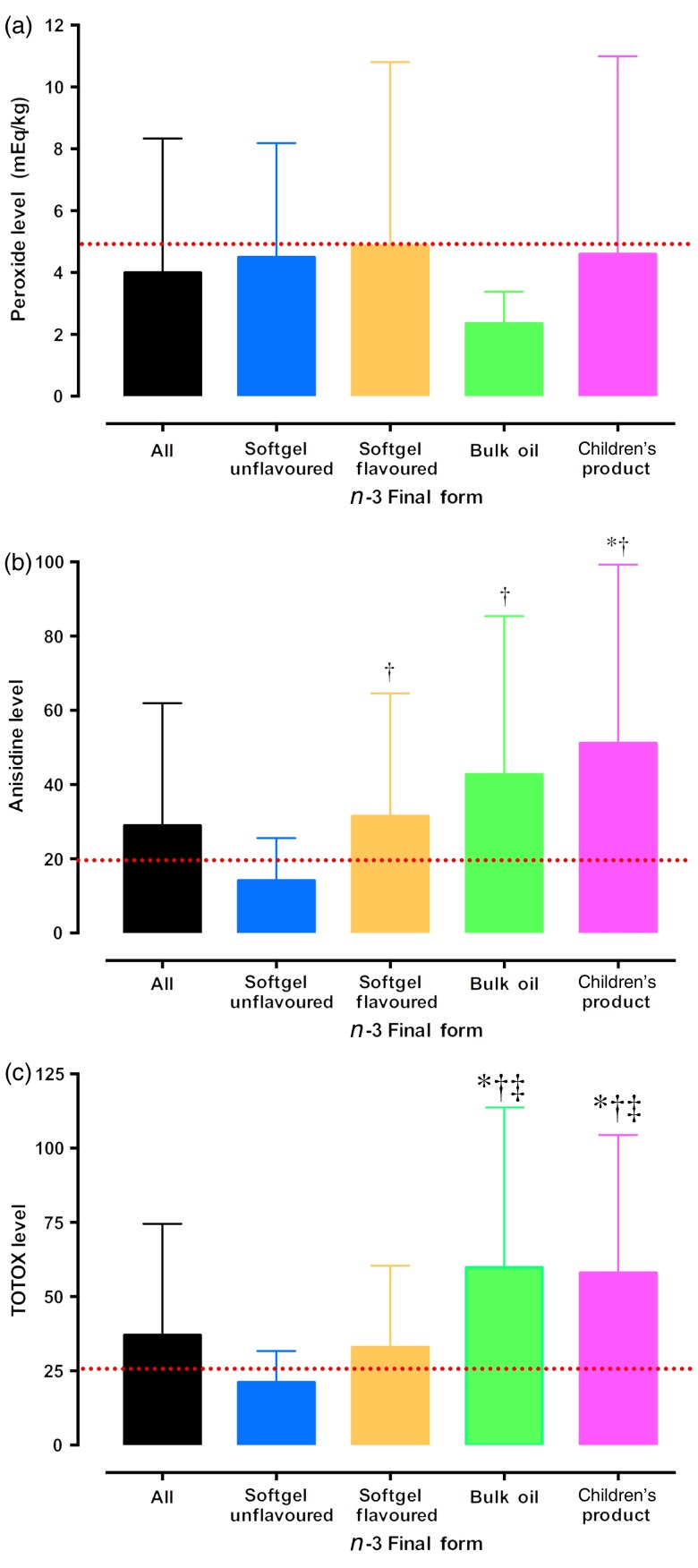
Peroxide (a), anisidine (b) and total oxidation (TOTOX) (c) levels of over-the-counter *n*-3 nutritional supplements available in Canada grouped by their final consumer form. * Mean value was significantly different from that for all *n*-3 nutritional supplements (*P* < 0·05). † Mean value was significantly different from that for softgel unflavoured *n*-3 nutritional supplements (*P* < 0·05). ‡ Mean value was significantly different from that for softgel flavoured *n*-3 nutritional supplements (*P* < 0·05). The dotted lines represent the safety limits (peroxide, <5 mEq/kg; anisidine, <20; and TOTOX, <26) recommended by the Global Organization for EPA and DHA Omega-3s (GOED), the Council for Responsible Nutrition (CRN) and the International Fish Oil Standards (IFOS)^(^^19^^–^^22^^)^.

Fig. 1 displays the mean peroxide, anisidine and TOTOX values for *n*-3 PUFA supplements grouped by product final consumer form. While no significant differences in peroxide levels were observed between product final consumer forms (*P* > 0·05; Fig. 1(a)), group comparisons indicated that anisidine levels for flavoured softgels, bulk oil and children's products were significantly higher than unflavoured softgels (*P* < 0·001; Fig. 1(b)). Additionally, it was observed that children's products had significantly higher anisidine levels than all *n*-3 PUFA nutritional supplements combined (*P* < 0·001). Similarly, group comparisons revealed that bulk oil and children's products had significantly higher TOTOX values than unflavoured and flavoured softgel products and all *n*-3 PUFA supplements combined (*P* < 0·001; Fig. 1(c)).

#### Oil source comparisons

**Fig. 2.**
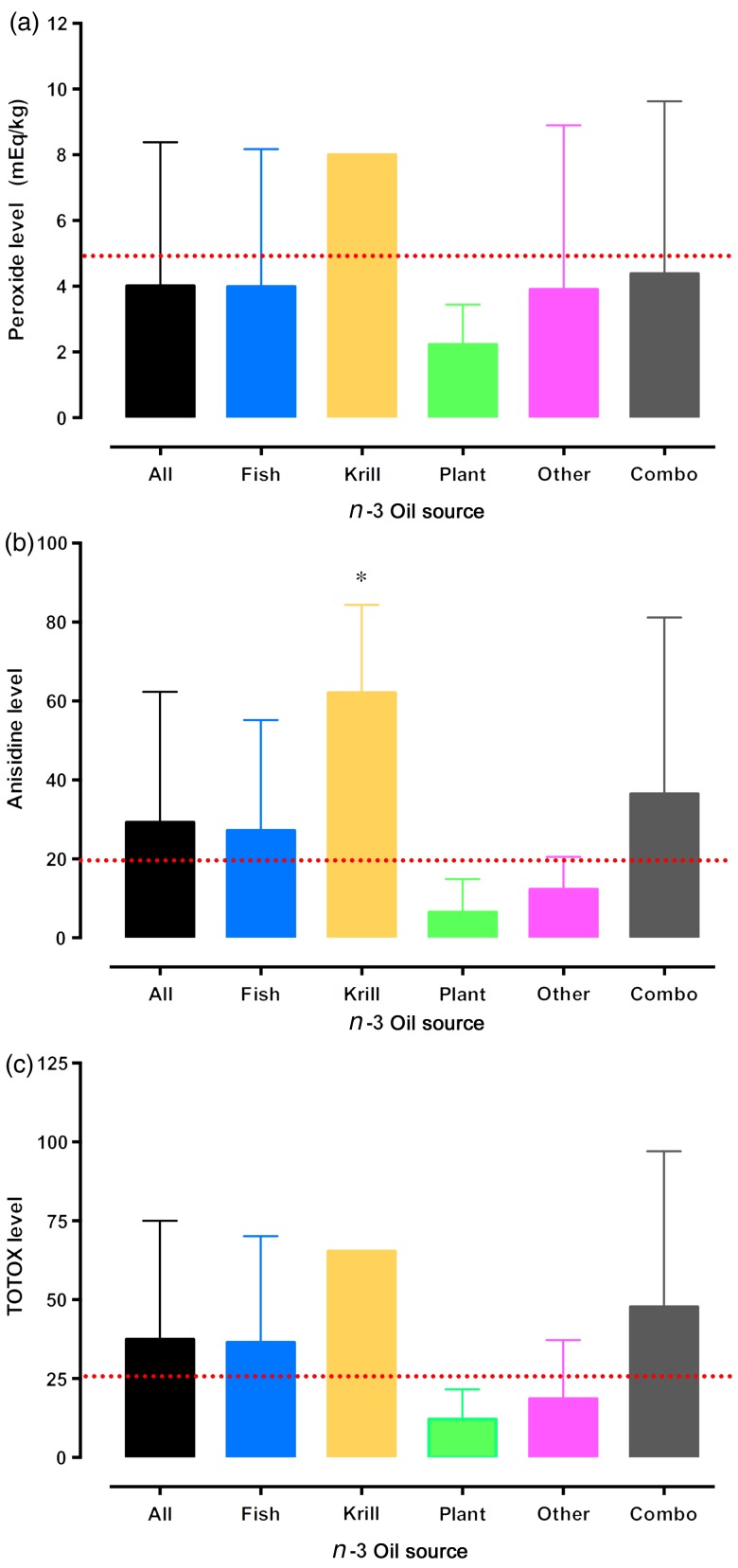
Peroxide (a), anisidine (b) and total oxidation (TOTOX) (c) levels of over-the-counter *n*-3 PUFA nutritional supplements available in Canada grouped by product oil source. No error bars are presented for peroxide (a) and TOTOX (c) levels in krill products because mean values were derived from a single product. * Mean value was significantly different from that for plant-based oil source *n*-3 nutritional supplements (*P* < 0·05). The dotted lines represent the safety limits (peroxide, <5 mEq/kg; anisidine, <20; and TOTOX, <26) recommended by the Global Organization for EPA and DHA Omega-3s (GOED), the Council for Responsible Nutrition (CRN) and the International Fish Oil Standards (IFOS)^(^^19^^–^^22^^)^. Combo, combination.

Fig. 2(a) displays the mean peroxide values for *n*-3 PUFA supplements grouped by oil source. Although eight krill oil *n*-3 PUFA supplements were included in the analyses, the peroxide level from only one krill oil product was successfully ascertained using the AOCS methods. No significant differences in peroxide levels were observed between *n*-3 PUFA supplements derived from different oil sources. For anisidine assessments, it was observed that krill oil *n*-3 PUFA supplements had significantly higher levels of anisidine than plant oil supplements (*P* < 0·05, Fig. 2(b)); however, no significant differences in anisidine levels were observed between all other oil source groupings (*P* > 0·05; Fig. 2(b)). Similar to the peroxide assessments, the TOTOX value from only one krill oil product was successfully ascertained using the AOCS methods. No significant differences in TOTOX levels were observed between *n*-3 PUFA supplements derived from different oil sources (*P* > 0·05, Fig. 2(c)).

#### EPA + DHA concentration comparisons

**Fig. 3.**
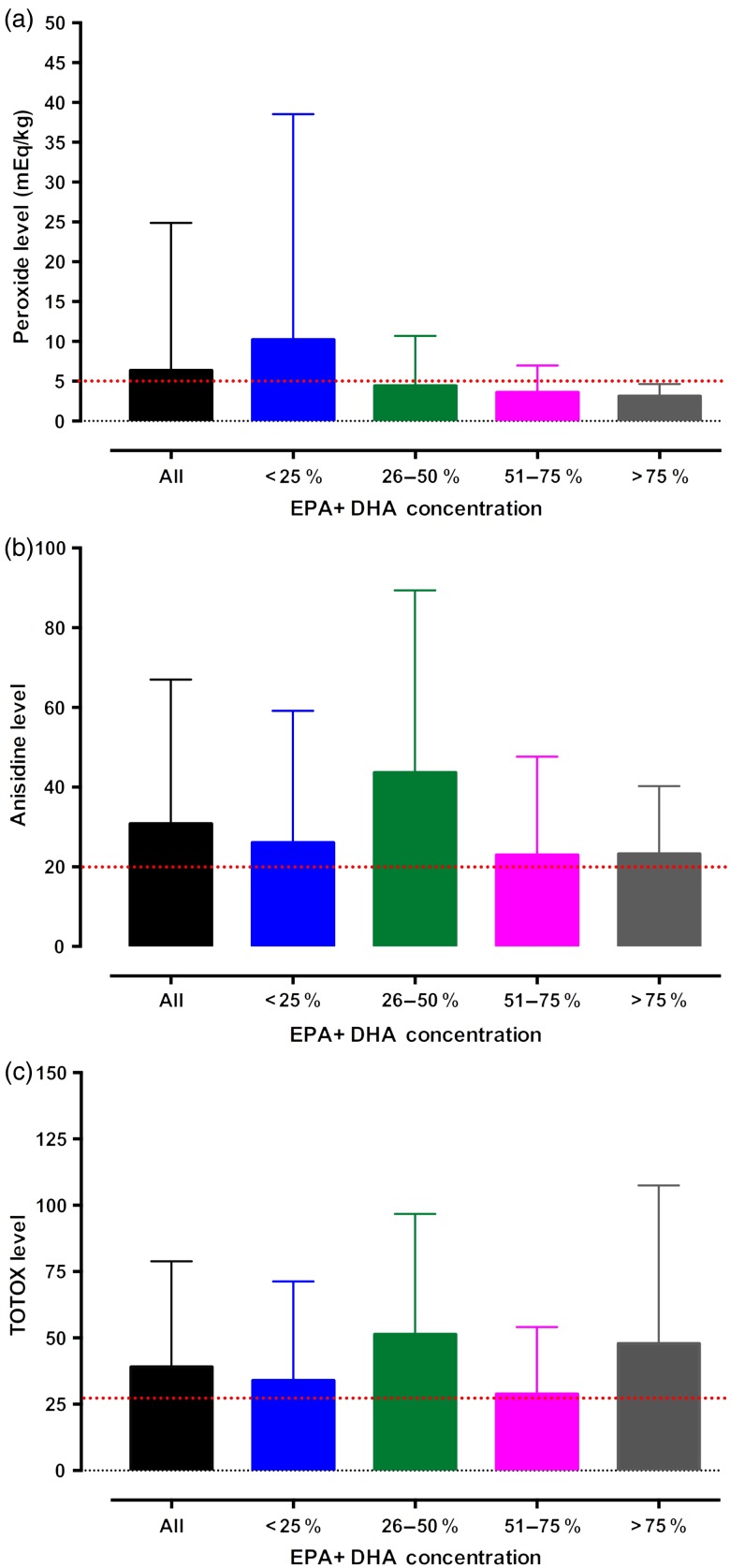
Peroxide (a), anisidine (b) and total oxidation (TOTOX) (c) levels of over-the-counter *n*-3 PUFA nutritional supplements available in Canada grouped by the percentage of EPA + DHA per 1000 mg of total product. Values are means, with standard deviations represented by vertical bars. The dotted lines represent the safety limits (peroxide, <5 mEq/kg; anisidine, <20; and TOTOX, <26) recommended by the Global Organization for EPA and DHA Omega-3s (GOED), the Council for Responsible Nutrition (CRN) and the International Fish Oil Standards (IFOS)^(^^19^^–^^22^^)^.

Fig. 3 displays the mean peroxide, anisidine and TOTOX values for EPA + DHA concentration quartiles. There were no statistically significant differences observed in the peroxide, anisidine or TOTOX levels between concentration quartiles.

### Products approaching limits

**Fig. 4.**
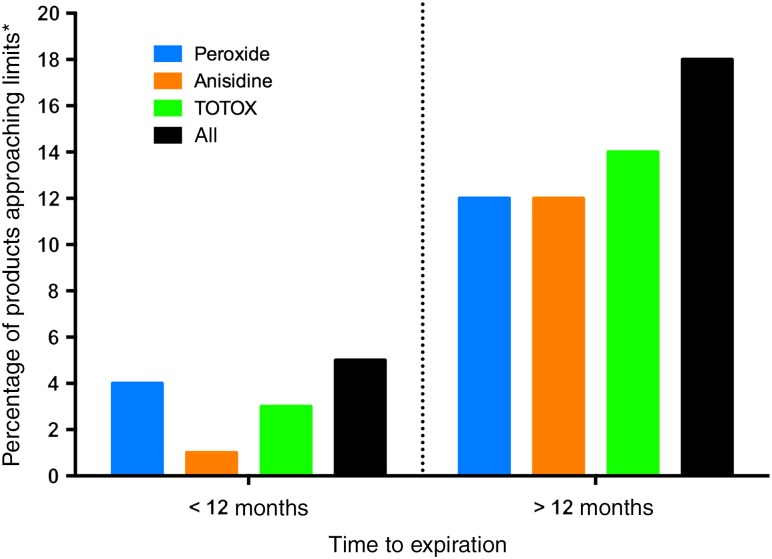
Percentage of total products tested that were approaching, but did not exceed, the voluntary safe limits. * Products were considered to be approaching the limits if they were exceeding 75 % of the voluntary safety limits for peroxide, anisidine and total oxidation (TOTOX) levels recommended by the Global Organization for EPA and DHA Omega-3s (GOED), the Council for Responsible Nutrition (CRN) and the International Fish Oil Standards (IFOS)^(^^19^^–^^22^^)^. The All group consists of products approaching the limit in at least one of all the markers of oxidation.

Fig. 4 displays the proportion of products approaching the voluntary safety limits with less than or greater than 1 year to expiration. It was observed that in addition to the number of products that failed the voluntary limits, a further 4, 1 and 3 % of all the products tested were approaching the voluntary safety limit for peroxide, anisidine and TOTOX, respectively, and had less than 1 year to expiration. In contrast, beyond those seen to exceed the voluntary limits, a further 12, 12 and 14 % of all the products tested were approaching the voluntary safety limits for peroxide, anisidine and TOTOX, respectively, and had more than 1 year to expiration. Additionally, outside of the products that failed at least one of the voluntary safety limits, a further 18 % of the products tested were considered to be approaching at least one of the limits for oxidation (e.g. peroxide, anisidine or TOTOX) and had more than 1 year until expiration (Fig. 4).

## Discussion

The aim of this study was to examine the oxidation status of North American *n*-3 PUFA nutritional supplements commercially available over the counter in Canada by determining their peroxide, anisidine and TOTOX levels using established testing procedures against voluntary safety recommendations. It was observed that 50 % of the OTC *n*-3 PUFA products tested failed at least one of the three voluntary safety standards recommended by GOED, CRN and IFOS and another 18 % were approaching these limits with 1–3 years until their expiration. These high rates of oxidation occurred in *n*-3 PUFA nutritional supplements despite the majority of products (89 %) containing some form of antioxidant. This is the first study, to our knowledge, to investigate the primary and secondary oxidation products (peroxide, anisidine and TOTOX) of OTC *n*-3 PUFA supplements available in Canada.

There are currently no set standards for assessing the primary and secondary oxidation status of OTC *n*-3 PUFA nutritional supplements; however, voluntary international industry standards currently recommend that peroxide, anisidine and TOTOX values below 5 mEq/kg, 20 and 26, respectively, should be employed for fish oils supplements to be considered safe for consumption^(^^19^^–^^22^^)^. In the present study, 171 products, consisting of forty-nine brands, available as OTC *n*-3 supplements were selected for analysis. From these, nearly 20 % could not be assessed by the AOCS methods used to determine either primary and/or secondary oxidation status. This was particularly evident for krill oil-based products as only one of the eight products was successfully tested for peroxide and subsequently TOTOX. These supplements’ oxidation levels could not be ascertained due to colours and/or additives interfering with end-point determinations following AOCS titration procedures. Although the AOCS methods are widely endorsed and accepted for the assessment of primary and secondary oxidation products, its reliance on visual detection makes it insensitive to detect peroxide and anisidine levels in a number of *n*-3 PUFA supplements with deep colour variations, as prominently seen in krill products. There are a number of commercially available alternatives for the assessment of peroxide, anisidine and other secondary oxidation measures (e.g. SafTests), such as malonaldehydes and alkenals. No comparative studies, to our knowledge, have been conducted to assess whether these alternatives provide greater sensitivity and can be used to supplement current testing procedures to provide a better illustration of lipid oxidation. Further research using alternative methods for assessing primary and secondary oxidation in Canadian *n*-3 PUFA products is thus warranted.

Regardless of the limitations of the AOCS methods, 139, 160 and 134 *n*-3 PUFA supplements were successfully assessed for peroxide, anisidine and TOTOX, respectively. For primary oxidation, it was observed that 17 % of the supplements successfully tested had peroxide values above 5 mEq/kg. This is lower than those reported in previous studies on OTC *n*-3 PUFA supplements^(^^23^^–^^29^^)^, where between 31 and 84 % of *n*-3 PUFA supplements exceeded voluntary safety limits for markers of primary oxidation. The lower peroxide levels observed in the present study's OTC products tested may be a result of a number of factors. First, efficient testing procedures were employed in the present study to reduce the exposure of supplements to oxidising conditions. All supplements were analysed within 30 min of extraction and refrigerated in clean sterile glass containers under an N_2_ blanket when not being analysed. Second, previous studies investigated oxidation levels in marine fish oils only and did not include other derivatives of *n*-3 PUFA (e.g. plant, seal, etc.). Plant oil-based *n*-3 PUFA tend to have shorter carbon chain *n*-3 PUFA, which may be less readily oxidised than the longer *n*-3 PUFA in fish oil products, and their inclusion in the present analyses may have reduced the number of product failures in the present study. Third, flavour additives may have influenced peroxide levels. Though there are currently no other studies documenting the primary and secondary oxidation of flavoured *n*-3 PUFA supplements, flavour modifiers such as sweet solutions/herbs and botanicals may aid in the breakdown of oxidative products. Fourth, the oxidation cycle may be more advanced in the products tested, where the primary oxidation products (e.g. peroxides) may have already progressed into the secondary oxidative stages. Thus, evidence of low peroxide values are consistent with both minimal and severe levels of oxidation, showcasing that primary oxidation may not be the paramount measure of oxidation status as solely employed by previous studies^(^^23^^–^^27^^,^^29^^)^. The assessment of secondary oxidative products serves to complement the primary oxidative products and warrants consideration when evaluating oxidative status of *n*-3 PUFA supplements, but has only been employed by a few studies^(^^28^^)^. The higher failure rates observed for anisidine and TOTOX in the present study may be indicative of this advanced oxidative progression. Of the *n*-3 PUFA supplements successfully tested for anisidine and TOTOX, 41 and 39 % had levels exceeding the voluntary limits. These percentages parallel previous observations by Albert *et al.*^(^^28^^)^, who observed that 25 and 50 % of the fish oil products available in New Zealand failed anisidine and TOTOX limits recommended by the GOED. While these higher levels support the notion that these products may be further along the oxidative process, flavour additives may also influence these measures. The contributory influence of flavour and other additives on oxidation determination was further evident when supplements were separated by product final consumer form (Fig. 1). It was observed that *n*-3 PUFA supplements containing flavour additives, whether as a capsule or bulk oil format, had significantly higher anisidine and TOTOX levels than unflavoured OTC supplements. Flavour additives, especially citrus flavourings, do contribute in anisidine testing, leading to elevated values, because they often consist of aldehyde-like chemical structures. While the current AOCS testing procedures are not designed to indicate the source of the oxidative products, rather their presence and abundance in the supplements tested, these findings do suggest that products containing flavour additives are associated with higher levels of secondary oxidative products. Whether these higher values are due to real oxidative degradation of *n*-3 PUFA or artificial due to flavour interference, are beyond the AOCS testing procedures. Yet, these higher oxidation levels may be of concern to consumers given their associations with pathogenic mechanisms in chronic diseases^(^^17^^,^^18^^)^. Moreover, this is especially concerning given that nearly 80 % of the children's products tested in the present study contained flavoured additives and were observed to have significantly higher levels of anisidine and TOTOX compared with all other *n*-3 PUFA supplements tested. Since there are currently no procedures, that the authors are aware of, that can adequately differentiate the independent contributions of oxidative products from oil sources and flavour additives, regulatory bodies should consider exercising greater control of permissible flavours and/or additives in *n*-3 PUFA supplements, or consider the design of novel testing procedures that can better assess the oxidative status of all *n*-3 PUFA supplements available for OTC consumption, including ones containing flavours.

The concentration of *n*-3 fatty acids found in *n*-3 PUFA supplements has recently been argued to be a critical component to a supplement's cardioprotective characteristics^(^^13^^,^^32^^–^^34^^)^. Given that concentrated products have higher quantities of *n*-3 PUFA, it can be reasoned that at elevated concentrations, lipid peroxidation may develop more readily and cascade to secondary oxidative products quicker once initiated, rendering these products more susceptible to oxidative products. The present study addresses this supposition. It was observed that there was no significant difference in markers of oxidation between products quartiled by EPA + DHA concentrations, showcasing that the concentration of EPA + DHA was not associated with the oxidative levels detected. These results complement recent observations by Albert *et al*.^(^^28^^)^ who report that the amount of EPA + DHA claimed on *n*-3 PUFA in OTC supplements was not a significant predictor of peroxide, anisidine or TOTOX markers in New Zealand and Australian products.

Consumers should also be concerned with the potential stability of their *n*-3 PUFA supplements as they are all susceptible to oxidation. While 50 % of the products tested in the present study failed at least one of the voluntary recommended limits, the remainder were seen to conform within these limits; however, this does not prevent these products from developing elevated levels of oxidation within their suggested shelf life. Of the products that were witnessed to be below the voluntary limits, eighty-two were more than 1 year from expiration. This lengthy shelf life may leave these ‘safe’ products susceptible to further oxidation prior to their end of use. Kolanowski *et al*.^(^^25^^)^ recently observed that the peroxide values of OTC encapsulated fish oil *n*-3 PUFA supplements available in Poland that were maintained in controlled conditions (e.g. at 20°C, in air-tight containers, with limited exposure to light) developed peroxide levels 20 % higher than their initial values within 22 d of storage. These authors concluded that with longer storage, the safety and quality of *n*-3 PUFA fish oils might become questionable. Thus, a peroxide level of 3·75 mEq/kg could rapidly rise to exceed the voluntary safety limits in a short period of time. In the present study the number of supplements that were approaching the voluntary limits (i.e. exceeding 75 % of the recommended limits) was also investigated with the presumption that these products could potentially reach these voluntary limits given sufficient storage time. Of the products considered to be within the safety limits, it was observed that thirty-nine were approaching at least one of the voluntary limits, of which thirty were 1 to 3 years from expiration. This suggests that an additional 18 % of products tested were approaching the voluntary safety limits and were more than 1 year away from their stated dates of expiration. Though this rate of oxidation is dependent on numerous factors such as the storage material and exposure to light and air, it does propose that 68 % of the products tested may exceed the voluntary safety limits and expose consumers to higher oxidative products well before their dates of expiry.

The high oxidation status of North American OTC *n*-3 PUFA supplements tested in the present study may be attributed to the lax regulatory requirements for natural health products sold in Canada. All non-prescription OTC *n*-3 PUFA supplements sold in Canada are controlled by the Natural Health Products Regulations. All *n*-3 PUFA supplements sold in Canada must have a product license, meet good manufacturing practice, and provide evidence of safety and efficacy. Within these regulations, however, there are no standards outlining the assessment of oxidation status as a safety requirement of OTC *n*-3 PUFA supplements or testing of products post-final formulation. This oversight is probably due to insufficient data identifying the ideal procedures and safety limits that would be applicable to all *n*-3 PUFA supplements; however, voluntary industry standards for fish oils have been proposed by international organisations^(^^19^^–^^22^^)^ and served as the foundation for the present study. The applicability of these voluntary limits, designed specifically for fish oil products, onto other *n*-3 PUFA supplements that may contain plant, krill and other source oils warrants consideration. Given that all *n*-3 PUFA supplements are highly susceptible to undergo oxidation due to the large number of double bonds within the fatty acid chain^(^^15^^)^, standardised procedures and limits are necessary to determine the safety and efficacy of these products regardless of the source oil used to derive these fatty acids. Additionally, these standards should be applicable to final end products, not merely to the individual ingredients prior to final consumer formulation. The encapsulation and bottling processes allow for oxidation of *n*-3 PUFA, if appropriate precautions are not adhered to. The present voluntary limits recommended by international bodies pertain to the source oil only, before encapsulation or bottling, and not the final consumer form, which may be consumed after these additional potentially oxidative processes. Furthermore, as is evident from the present study, colours, flavours and other additives may interfere with or sufficiently affect the detection of oxidation products in *n*-3 PUFA supplements and should be tightly regulated to either conform to the voluntary standards or undergo testing procedures that adequately ascertain their safety and efficacy. It should be noted that *n*-3 fatty acids are delivered in a number of alternative forms (e.g. ethyl esters, TAG, re-esterified TAG). These may further affect the oxidation status of the OTC supplements and were not considered in the present study. Future research investigating the role of *n*-3 fatty acid form on oxidative status warrants consideration and would supplement the findings of the present study. Regardless, the variability in oxidative status seen between different studies continues to suggest that the oxidation levels in *n*-3 PUFA supplements can vary from country to country. With most countries having no governing body to regulate *n*-3 PUFA supplements, the prudence of manufacturers to test each batch of final products is not vigilantly enforced. This may lead to supplements with high levels of primary and secondary oxidation products being purchased by unaware consumers.

Finally, based on the present study results and analysis, from a consumer point of view, an ideal *n*-3 PUFA nutritional supplement should be one that is highly efficacious in alleviating the *n*-3 PUFA deficiency that exists in North America, be safe as per defined oxidative safety limits and be testable in their final consumer form using established testing procedures, as used in the present study, to ensure both their efficacy and safety. It is already established that a highly concentrated *n*-3 PUFA formulation may be the most effective in correcting an *n*-3 PUFA deficiency^(^^13^^,^^32^^–^^34^^)^ in as little as 4–8 weeks, but the present data speak to the safety and testing aspects of an ideal *n*-3 PUFA nutritional product for consumer usage. Unflavoured softgels containing highly concentrated fish oil-based *n*-3 PUFA appear to sufficiently meet the criteria described above and should be considered as an ideal *n*-3 PUFA product by consumers. These types of nutritional supplements can be adequately tested using current established procedures, comply with internationally recognised safety limits, and deliver the benefits associated with *n*-3 PUFA nutritional supplementation.

### Conclusions

In summary, oxidative status in a large sample of OTC *n*-3 PUFA nutritional supplements was determined including products with diverse delivery forms and formulations. Of the *n*-3 PUFA nutritional supplements tested, 50 % of them failed at least one of the voluntary safety standards recommended for primary and secondary oxidation and TOTOX. Oxidative safety was further investigated to evaluate the contributions of formulation, delivery form and time to expiry using current standardised AOCS testing procedures. Based on these parameters, encapsulated, unflavoured fish oil *n*-3 PUFA nutritional supplements appear as the safest and readily testable consumer product. Present oxidation safety recommendations endorsed by international agencies are applicable to fish oil products only. Their scope needs to be extended to all *n*-3 PUFA, regardless of delivery form or formulation. Oxidative safety limits need to be introduced by regulatory bodies to ensure the safety, compliance, testability and efficacy of all *n*-3 PUFA nutritional supplements.
